# Artificial oil bodies: A review on composition, properties, biotechnological applications, and improvement methods

**DOI:** 10.1016/j.fochx.2023.101109

**Published:** 2024-01-04

**Authors:** Ruhuan Yuan, Jianying Liu, Ruchika Hansanie Ukwatta, Feng Xue, Xiaohui Xiong, Chen Li

**Affiliations:** a College of Food Science and Light Industry, Nanjing Tech University, 30 Puzhu South Road, Nanjing, 211816, PR China; b School of Pharmacy, Nanjing University of Chinese Medicine, 138 Xianlin Road, Nanjing 210023, PR China

**Keywords:** Artificial oil bodies, Composition and Structure, Reconstitution methods, Controlling factors to stability, Applications, Improvement measures

## Abstract

In order to simulate the structure of natural oil body, artificial oil bodies (AOBs) are fabricated by the integration of oleosins, triacylglycerols (TAGs) and phospholipids (PLs) *in vitro*. Recently, AOBs have gained great research interest both in the food and biological fields due to its ability to act as a novel delivery system for bioactive compounds and as a carrier for target proteins. This review aims to summarize the composition and the preparation methods of AOBs, examine the factors influencing their stability. Moreover, this contribution focusses on exploring the application of AOBs to encapsulate functional ingredients that are prone to oxidation as well as improve efficiency involved in protein purification, renaturation and immobilization by reducing the complex steps. In addition, the improvement measures to further enhance the stability and efficacy of AOBs are also discussed. The application of AOBs is expected to be a big step towards replacing existing bioreactors and delivery systems.

## Highlights

• AOBs can be constructed by OBPs, PLs, and TAGs.
• The stability of AOBs depend on pH, constituent proportions, and ultrasonic power.
• AOBs are used for purification, renaturation and immobilization of fusion protein.
• AOBs provide an effective delivery system for drugs and bioactive compounds.
• Functional properties of AOBs can be improved by altering the fabrication process.

## Introduction

Naturally occurring oil bodies derived from plants, with a micron-size of 0.3–0.4 μm, are spherical structures comprised of TAGs, PLs, and peculiar membrane proteins (Zhang et al., 2022). The predominant membrane proteins are oleosins, supplemented by small quantities of caleosins and steroleosins. The stability of oil bodies is maintained by oleosins along with caleosins and steroleosins, which primarily endow oil bodies with important surfactant properties to avoid coalescence (Abdullah et al., 2020). Oleosins contain three structural domains: an *N*-terminal; a C-structural domain; and a central hydrophobic domain. Among them, the central hydrophobic structural domain is embedded into the oil body core by amino acid residues of β- sheets, which plays a key role in the environmental stability of natural oil bodies (Sen et al., 2022). Whereas, the hairpin structure formed by the central hydrophobic structural in natural oil body domain makes it difficult to encapsulate hydrophobic compounds, limiting its application areas (Sun et al., 2020). Thus, AOBs reconstructed by TAGs, PLs, and membrane proteins in a certain proportion have been paid more attention due to their attractive advantages. The stability, loading capacity, and biocompatibility of AOBs can be enhanced by adjusting the ratio of lipids to proteins and modification of oleosin proteins, leading to better control of the drug release and loading (Vargo et al., 2015). AOBs can also serve as a carrier for the expression of recombinant proteins, enzyme immobilization and as delivery of hydrophobic molecules (Chiang et al., 2016). High concentrations (1.25 %-10.0 %, w/v) of AOBs can induce a certain degree of cytotoxicity, whereas low concentrations (0.56 %-1.0 %, w/v) are essentially non-toxic, exhibiting great safety and favorable biocompatibility *in vivo* (Ding et al., 2021). In addition, the oil phase and surface oleosin provide AOBs with a lower digestion rate in gastric fluid, but a gradually increasing rate in intestinal fluid (Sun et al.,2022). These characteristics enable sustained release of bioactive molecules, making it suitable for pharmaceutical and food applications.

**Fig 1:**
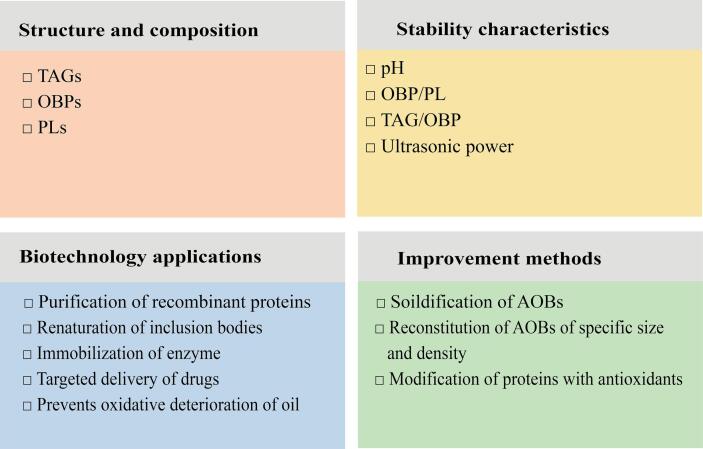
An overview diagram of AOBs including structure and composition, stability characteristics, biotechnology applications, and improvement methods.

This review summarizes recent research findings on AOBs, focusing on their composition and the factors influencing their stability. Particular attention is given to the potential application of AOBs in the field of biotechnology and food industry, and measures for their improvement (Fig. 1).

## Composition and structure of AOBs

**Fig 2:**
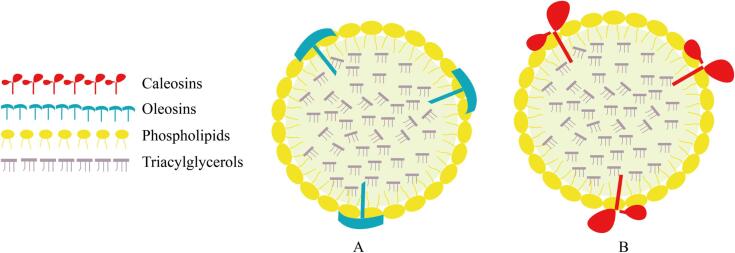
The structure of AOBs. (A) Construction of AOBs with oleosins. (B) Construction of AOBs with caleosins. The components of the illustration are not on-scale.

Similar to natural oil bodies, AOBs are spherical structures with a hydrophobic core composed of triacylglycerols surrounded by a protein-phospholipid monolayer. In general, AOBs primarily consist of PLs, proteins, and TAGs, and do not contain bioactive components found in natural oil bodies, such as isoflavones and tocopherols (Fisk and Gray, 2011, Zaaboul et al., 2018). Currently, membrane proteins utilized for constructing AOBs include oleosins and caleosins. Normally, oleosin is chosen for applications in food systems because it accounts for 80–90 % of the OBP content (Zaaboul et al., 2022), which is easy to extract, while calesin is mainly used in biotechnology field. (Fig.2).

### Oil body proteins (OBPs)

OBPs are amphiphilic topological proteins including oleosin, caleosin, and steroleosin, which are embedded in PL monolayer and can stabilize oleosomes through steric hindrance and electronegative repulsion (Li et al., 2022). Among the three proteins presented in the oil body, only oleosin and caleosin can play a role in maintaining the structural stability of AOBs due to their similar structural domains, in which 76 and 32 residues of the central hydrophobic domains can be used to anchor the oil bodies, respectively (Liu et al., 2009). Oleosins with molecular weight around 15–30 kDa are alkaline proteins that contain seven oleosin isoforms divided into H- and l-isomers, both of which can be used to stabilize the AOBs but l-isomer is more stable than H-isomer (Tai et al., 2002, Zaaboul et al., 2022). Caleosin accounts for a relatively low percentage of total OBP. It has a hydrophilic *N*-terminal and a hydrophobic C-terminal that accounts for 85 % of the total size and covers more surface area of the oil body than oleosin (zaaboul et al., 2022). Caleosin-stabilized AOBs are more thermally stable and smaller in size, which is beneficial in biotechnological applications (Chen et al., 2004, Sun et al., 2021).

### TAGs

TAGs, which provide energy during the germination of plant seeds, are the main component of the lipid core. TAGs accounting for about 92.5 % of the oil body content, along with small amounts of waxes and sterol esters (Shimada et al., 2008). Because of the hydrophobic nature of TAGs, the non-polar ends of monolayer PLs can wrap which to form a stable spherical structure. Vegetable oils include olive oil, soybean oil, and sesame oil are commonly used when reconstituting AOBs. In addition, mineral oils have also been attempted to construct AOBs but are found to be less stable than AOBs reconstituted with vegetable oils. (Chiang et al., 2011, Chiang et al., 2012).

### PLs

PLs associated with unique membrane proteins are the main components that make up the monolayer surrounding and stabilize the lipid core, which consist of phosphatidylcholine (PC), followed by phosphatidylethanolamine (PE), phosphatidylinositol (PI), and phosphatidylserine (PS). Phosphatidylcholine accounts for approximately 41.2 % to 64.1 % of total PL and is the most representative type of PL in oil bodies (Sen et al., 2022). Due to their distinctive amphiphilic nature, PLs can effectively maintain the stability of AOBs when combined with OBPs (Karefyllakis et al., 2019). The polarity of phospholipids has been reported to have an effect on the stability of AOBs during reconstitution of AOBs. For instance, Bourgeois et al. (2019) demonstrated that PLs with different polar heads can cause distinct protein conformations by altering protein charge, which can have an impact on the stability of the binding of OBPs to the PL membrane. Besides, zwitterionic PLs exhibit only slight interactions with oil proteins, while anionic PLs can enhance the interaction with oil proteins and improve their physicochemical stability.

## Reconstitution methods of AOBs

### Preparation of OBPs

#### Extraction of OBPs from oilseeds

The membrane proteins used to construct AOBs can be extracted directly from natural plant seeds. There are two main steps in extracting OBPs from plant seeds. The first is to extract the oil bodies from the oil seeds and the second is to isolate the OBPs from the oil bodies. Ding et al. (2020) first extracted the oil bodies by aqueous solvent method, washed more exogenous proteins by centrifugation three times in an alkaline environment, then isolated the OBPs from the oil bodies, centrifuged them after degreasing with acetone, collected the precipitate by centrifugation twice. Then, the remaining acetone is removed in a nitrogen blowing apparatus and the separated OBPs are lyophilized and dried for use. The key step in this method is degreasing, but the organic solvent used for degreasing can have a negative impact on the protein. Ntone et al. (2020) have improved the method of extracting OBPs by further concentrating and filtering using ultrafiltration, which allows the extraction of OBPs under mild conditions and reduces the negative effect of organic solvents on the properties of the extracted proteins.

#### Expression of OBPs using *e. Coli*

Apart from extraction from oil seeds, OBPs can also be expressed in bacterial systems (Bhatla et al., 2010), due to the high hydrophobicity of oleosin's central hydrophobic structural domain facilitating oleosin gene isolation from plant seeds (Capuano et al., 2007). Compared with obtaining OBPs from plant oil seeds, purification and expression of oleosin and caleosin by bacteria can not only increase the yield and improve the production efficiency but also change the size of AOBs by modifying the OBP genes. Peng et al. (2007) induced the target plasmids by PCR and produced five oleosin constructs with different residue numbers through deletion of the central hydrophobic structural domain. Five different oleosins were then overexpressed by *E. coli* and reconstituted with TAGs and PLs to form AOBs. It is found that when the length of hydrophobic region in the center of oleosins is greater than 36 residues, its structural stability was similar to that of the natural oil body. However, when the length of the central hydrophobic region is less than half of the original, AOBs were prone to aggregation. In addition, Chang et al. (2013b) also cloned the oil body calmodulin gene from sesame into a PET29a (+) plasmid vector, transformed in *E. coli* BL21 and overexpressed in the phage polymerase expression system. Finally, *E. coli* was crushed and caleosin was extracted for recombinant AOBs.

### Construction of AOBs by ultrasound

**Fig 3:**
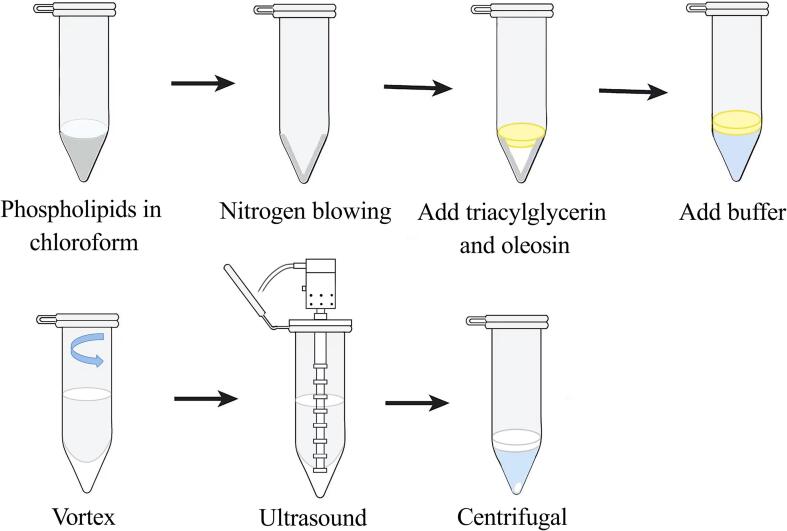
Flowchart depicting AOBs construction.

At present, the method of constructing AOBs is very mature, and the main method of reorganization is adopted by ultrasound. In the conventional method, PLs were first dissolved in chloroform. After thorough mixing, the excess chloroform was removed by a nitrogen blower to form a PL film in an Eppendorf tube. Subsequently, TAG and OBP were put into Eppendorf tubes in the established ratio and vortexed by adding sodium phosphate buffer solution. Next, the solution in the Eppendorf tube was sonicated by insertion of a titanium probe. Finally, the mixture was centrifuged and the upper white floating bands were the AOBs (Fig. 3) (Wang et al., 2016). Alternatively, TAGs, PLs, and OBPs can be mixed together in specific proportions and then sonicated with a titanium probe in an ice-water bath to reconstitute the AOBs (Hu et al., 2022).

## Controlling factors to stability of AOBs

In the process of reconstructing AOBs, how to improve their stability and make them better for practical applications is a hot topic of research. It is known that the stability of AOBs depends mainly on their size and surface properties. In recent years, it has been shown that many factors such as pH level, the content of TAGs, OBPs, and PLs as well as the processing techniques have an impact on the size and structure of AOBs.

### pH

For natural oil bodies, the pH affects the charge number and the exposure of the hydrophilic groups of the associated proteins on the surface of the oil bodies, which in turn affects the surface charge binding capacity and distribution as well as the particle size (Chen and Ono, 2010, Gao et al., 2022). Previous report suggested that when the pH deviates from the isoelectric point of OBPs in oil bodies, the absolute value of ζ potential increases with increasing surface charge. This results in a stronger electrostatic force between oil bodies, preventing their aggregation and thus enhancing stability (Qi et al., 2017).

Chiang et al. (2012) found that AOBs tend to agglomerate under acidic conditions through turbidity testing and transmission electron microscopy observations. However, under alkaline conditions, the oil body particles were more dispersed and independent. This may be due to the fact that the isoelectric point of the oil body membrane proteins is closer to acidic pH value, and the electrostatic repulsion on the surface of the oil body will be weakened in an acidic environment, leading to the agglomeration of AOBs with reduced stability. Chiang et al. (2011a) conducted a similar study with consistent results. Under acidic conditions, the AOBs would be possessed of larger size with lower stability than that in alkaline environment.

In the construction of AOBs, the pH values not only affect their stability but also have an impact on the activity of target products. Chiang et al. (2005) used AOBs for purification of nattokinase within a pH range of 7 to 9. They found that the yield and activity of nattokinase were higher when the pH value was set to 7.5. Therefore, the selection of pH for the construction of AOBs also requires a comprehensive consideration based on the encapsulated material or target products.

### OBP/PL

PL and OBP form the monolayer structure of the oil body works synergistically to maintain the stability of the oil body. Too little OBP or too much PL will lead to oil body with larger particle size, the ratio of PL to OBPs is therefore very important (Ho et al., 2014, Hu et al., 2009). Unlike natural oil bodies, the structural stability of AOBs can be modulated by altering the proportions of OBPs to PL. Different ratios of PL and OBPs have significant influence on the stability of AOBs.

Recently, Hu et al. (2022) investigated the changes of the secondary structure of interfacial protein and hydrogen bonding of the AOBs by governing the ratio of OBP and PL. It was found that with the increase of PL concentration, the change of secondary structure of interfacial proteins could cause significant changes in the stability of AOBs. When the random coils and β-turns in the interface protein increased, the stability of the AOBs will increase accordingly. When the ratio of OBP/PL was 0.5, the obtained AOBs had the highest content of random coils and β-turns, resulting in better emulsification, physical stability, and oxidation stability. In addition, although the number of hydrogen bonds in the interfacial protein molecules changes with the alteration of the OBP/PL ratio, this change does not affect the stability of the AOB. In a word, the mixing of PL and OBPs in different ratios mainly affects the stability of recombinant oil bodies by changing the secondary structure of interfacial proteins.

### TAG/OBP

The size of the AOBs can be adjusted by the ratio of TAG and OBP. By setting different ratios, Peng et al. (2003) found that the size of the AOBs decreases with the decrease of TAG/OBP ratio, and their stability increases accordingly. This may be due to the weaker electrostatic repulsion between the oil bodies as a result of the decrease in protein content. Chiang et al. (2010) also found the same pattern that the size of AOBs with high TAG/OBP ratio was larger and, on the contrary, smaller under optical microscopy.

### Ultrasonic power

Ultrasound is a common food processing technique that is safe and environmentally friendly (Awad et al., 2012). The mechanical and cavitation effects produced by high power ultrasound can cause the separation or aggregation of subunits, disrupting peptide bonds and affecting the structure and function of proteins (Arzeni et al., 2012). Currently, there are many studies on the effect of ultrasound on the particle size of proteins, some of which show that the particle size of proteins decreases after ultrasound treatment (Jun et al., 2020, O'Sullivan et al., 2016), while others show the opposite result (Chen et al., 2019, Chen et al., 2019). The possible reasons for this result are due to different power of ultrasound applied, where low-power ultrasound has been used to ensure the quality and safety of food, and high-power ultrasound, which can disrupt the interaction forces between protein molecules and lead to protein dissociation (Chen & Zhang, 2023).

Ultrasound is an essential condition in the construction process of AOBs. Li et al. (2019) studied the stability of AOBs by changing the ultrasound power and found that ultrasound treatment could change the secondary structure of proteins, which led to an increase in their emulsification ability. When the ultrasonic power is 400 W, the relative content of β-sheets structured protein in the AOBs increases, which extends the spatial structure and intensifies the degree of protein aggregation. Moreover, the particle size of the oil body increases, together with the decreased stability. When the ultrasonic power reduced to 200 W, the relative content of the α-helical structure of the OBP decreased, the particle size of the oil body is smaller, and the emulsion was more stable.

## Applications

**Fig 4:**
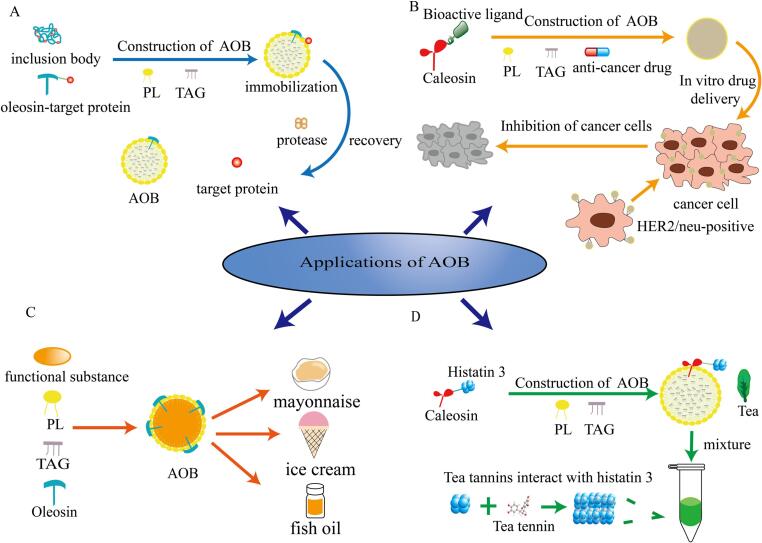
Applications of AOBs in the biotechnology and food field. (A): purification, renaturation and immobilization of proteins. Step 1: Inclusion body/ oleosin-target protein is constructed into AOB. Step 2: Inclusion body is spontaneously folded on the surface of AOB to restore activity, while the target protein is immobilized. Step 3: Add protease to recover the target protein on the surface of AOB. (B): Drug delivery process. (C): Encapsulation of functional substances. (D): Mechanism of sensory evaluation.

Unlike natural oil bodies, AOBs have a wider range of applications in biotechnology and the food industry. Various biological platforms have been developed using AOBs, including protein purification and expression systems, hydrophobic oral drug delivery systems, enzyme immobilization, and renaturation systems (Chang et al., 2013b). In the food industry, AOBs have gained widespread utilization for encapsulating hydrophobic nutrients due to their distinctive hydrophobic core, along with amphiphilic membrane proteins and phospholipids (Fig. 4).

### As a carrier for fusion proteins

Researches in the area of utilizing plant organelles as target gene vectors has evolved over decades. Currently, the development of plant expression systems is focused on directing the production of the desired protein towards plant-specific organelles, facilitating a more accessible isolation and purification process for the target protein (Franken et al., 1997). Oil bodies, as energy-storing organelles in plant seeds, possess the ability to produce target proteins, yet constructing AOBs can further exploit their potential. Both oleosins and caleosins in oil bodies can be used to construct fusion protein expression systems to accomplish the purpose of producing, renaturing, and immobilizing target proteins.

The steps to use AOBs to de-express the target protein vary depending on the purpose of the experiment. For purification of the target protein, it is necessary to cleave the OBP and the target protein with exonuclease and then centrifuge. Since the main component of AOB is TAG, which is highly hydrophobic and remains intact when subjected to external forces, it is easy to separate the AOB from the target protein by centrifugation. When using AOBs for the renaturation of inclusion bodies, the process is essentially similar to the purification step. During the process of reconstituting the AOBs, the inclusion bodies will spontaneously refold on the surface of which to restore the activity and function of the target proteins. Since the target protein is located on the surface of the AOBs, the separation step will be much convenient and fast. If the target product is an enzyme, it can be directly immobilized by using AOB and can be used without further centrifugation and separation, and the enzyme can also be recovered and reused by aqueous solution extraction after the enzymatic reaction is finished.

#### Purification of recombinant proteins

Several methods have been used for protein purification, such as affinity chromatography, gel filtration, and ion exchange chromatography. Among them, affinity chromatography is a relatively simple but expensive process, which requires the use of an affinity tag to fuse with the target protein, and then the recombinant protein is purified by elution in an affinity column (Schauer-Vukasinovic et al., 2002). Today, an ideal recombinant protein production system should be low cost and high safety. In contrast to bacteria and fungi as protein expression systems, which are not suitable for the production of many desired proteins due to differences in their protein and codon use, plants have a clear advantage as expression systems for purified recombinant proteins (Bhatla et al., 2010).

Plants are very safe protein expression systems because of their low potential for contamination by pathogens (Capuano et al., 2007). It has been shown that both leaves and seeds of plants can be used to produce recombinant proteins at low cost and at very high yields. However, leaves are not the most suitable route for expressing recombinant proteins due to their high content of proteases and polyphenols (Houdebine, 2009). However, the protein storage in seeds is more stable and less hydrolytic, and the expressed recombinant proteins can be preserved for a longer period, making them good recombinant protein carriers. So far, it has been found that the oil bodies in seeds are suitable as carriers of recombinant proteins, due to the recombinant proteins existing on the surface of the oil bodies could be easily separated by centrifugation (Ling, 2007).

**Fig 5:**
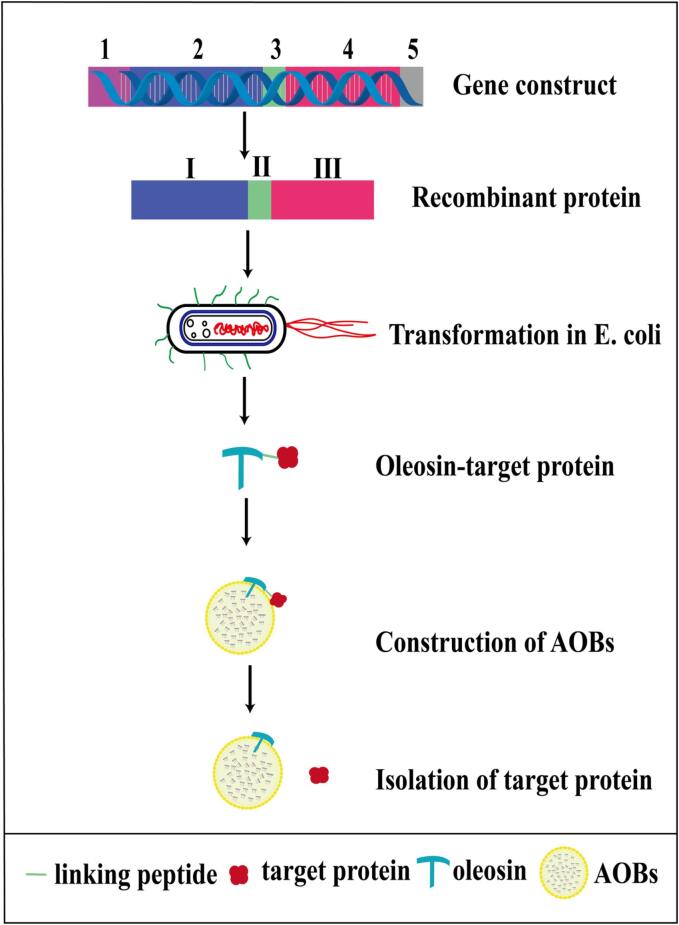
Recombinant and purification process of target proteins with oleosins. Step1: Oleosin-linker peptide-target protein gene fragment was constructed. Step2: Target protein is expressed by E. coli. Step3: Target protein is isolated and purified by recombinant AOBs. 1. Promoter; 2. Oleosin gene; 3. Gene encoding linking peptide; 4. Gene for target protein; 5. Terminator sequence. I. Oleosin; Ⅱ. Linking peptide; Ⅲ. Target protein.

Recombinant protein purification mainly involves the fusion of oleosins with heterologous proteins, which are expressed by *E. coli* and used to reconstitute AOBs (Fig. 5). Wherein, one of the most important steps is the separation of the heterologous proteins from the AOBs, which requires relatively expensive endopeptidases for cleavage, such as factor Xa and thrombin. Therefore, Peng et al. (2004) chose a relatively inexpensive endopeptidase, papain, in the production of Sesame cystatin using AOBs. It was confirmed that the strategy of using AOBs instead of papain affinity coupling columns for the production of sesame cystatin yielded higher economic benefits with the same purification efficiency. Similarly, when producing nattokinase by AOBs, (Chiang et al., 2005) tried to link the intrinsic peptide fragment to the OBP and nattokinase, and to achieve the separation of nattokinase by inducing self-shearing of the intrinsic peptide junction through temperature change. By using the intrinsic peptide instead of endopeptidase, both the cost and the limitation of AOBs in practical use can be reduced.

#### Renaturation of inclusion bodies

With the development of recombinant DNA technology, the use of bacteria (mainly *E. coli*) for mass production of target proteins has become popular. However, the process of reversibility varies with different proteins, and in general, the process of making proteins reversible is not only complex but also inefficient. Traditional renaturation methods have many steps, requiring adding denaturants, and removing excessive denaturants through gel filtration and dialysis (Fischer, 1994). Compared to this, the process of using AOBs to renature the inclusion bodies is more efficient. Choi and Chang (2009) designed the oleosin-ubiquitin-tagged IGF1 expression system for low-cost mass production of human insulin-like growth factor I (IGF-1) in *E. coli*, but the protein expressed was mainly in the form of insoluble inclusion bodies, which greatly reduced the IGF1 activity. However, by constructing AOBs from the expressed oleosin fusion protein with triacylglycerol and phospholipids, the renaturation steps can be simplified, which mainly includes three steps: solubilization, renaturation, and recovery. The insoluble oleosin fusion protein can be well solubilized in triacylglycerol and refolded on the surface of AOBs to restore activity, and then specific cleavage is performed by adding ubiquitin protease to obtain active IGF1. Compared with traditional methods, the use of AOBs provides a more convenient strategy to recover the protein by a simple centrifugation step and does not require the addition of denaturant, which guarantees higher production efficiency.

#### Immobilization of enzyme

Enzymes are biological macromolecules produced by living cells, which are essential proteins or RNAs, and they play a crucial role in many reactions due to their efficient catalytic activity. Today, enzymes have been found extensive application in various fields, including medical research, food processing, and waste utilization (Choi et al., 2015). Therefore, how to effectively reuse enzymes in practical applications is a challenge that needs to be overcome in all industries. A large number of studies have shown that the immobilization of enzymes is an effective method to solve this problem, but the stability and reusability of immobilized enzymes need to be further improved (Li et al., 2018). The catalytic efficiency of enzymes after immobilization is influenced by the immobilization carrier materials selected. Traditional immobilization carriers include natural polymers and graphene, etc. (Badoei-Dalfard et al., 2019, Patel et al., 2019). Although they can improve the catalytic activity of enzymes, they still have limitations such as low loading rate and specific pore size (Feng et al., 2022).

Nowadays, the AOB expression system provides a new idea of enzyme immobilization, due to the unique topology of oleosin, which has a lipophilic fragment embedded inside TAG and two amphiphilic arms located on the surface of oil body (Chiang et al., 2006). Therefore, the target enzyme gene can be fused with the amphiphilic N- or C-terminus of oleosin, expressed in *E. coli*, and then added with PL, TAG to reconstitute AOBs. The target enzyme fused to the oleosin will spontaneously refold on the surface of the AOBs to restore activity and achieve immobilization (Hung et al., 2008).

Bai et al. (2014) extracted the genomic DNA fragment of lipase from *Pseudomonas aeruginosa* to encode it and amplified the desired OBP sequence. Afterwards, the target OBP sequence was cloned into a specific site in the plasmid, and the DNA amplification sequence of the desired enzyme was digested with a specific restriction endonuclease and ligated with the plasmid containing the OBP gene fragment by DNA ligase. Finally, the immobilization of lipase was achieved by introducing the plasmid into *E. coli* to express the target fusion protein, which was used to construct AOB.

Tseng et al. (2014) immobilized D-Psicose3-epimerase on AOBs and found that the thermal stability and optimum temperature of the enzyme were improved, but the optimum pH value was reduced. The enzyme could still maintain 50 % viability after five cycles of treatment, and AOB as a solidification carrier could complete the refolding and immobilization of the enzyme simultaneously, which could significantly improve catalytic efficiency and reduce production time and cost, therefore is promising in practical applications.

### Targeted delivery of drugs

Targeted therapy is currently an effective way to treat cancer (Farokhzad & Langer, 2009). In the choice of targeting, biologically active ligands are usually combined with hydrophobic drugs for targeted delivery to cancer cells, releasing the drugs to produce an inhibitory effect so that damage to normal cells can be avoided (Cho et al., 2008). Nowadays, all well-known anti-cancer drugs on the market are hydrophobic, and direct oral or intravenous administration would result in low bioavailability (Lipinski et al., 1997). Therefore, carrier systems such as polymers, micelles, and liposomes have been developed to encapsulate hydrophobic drugs to enhance their utilization.

In the process of exploring new drug carriers, one must ensure that the size of encapsulated drug carriers is not too large, and that they are biocompatible and capable of targeted delivery to achieve efficient drug utilization to protect the survival of normal cells. AOBs meet these conditions because they are composed of natural proteins and lipids, and their nanoscale size not only improves pharmacokinetic properties but also enhances therapeutic effects (Ferrari, 2005). In recent years, more and more scholars have attempted to investigate the effectiveness of using AOBs as carriers to encapsulate hydrophobic drugs.

To deliver drugs by AOBs, an active targeting technique is generally adopted to construct new plasmid vectors from oleosin or caleosin with DNA fragments of specific bioactive ligands of target cells, which are produced and expressed using *E. coli*. The isolated fusion protein, vegetable oil, and PL were then mixed and additionally added with anti-cancer drugs for the reconstruction of AOBs, and the anti-tumor activity was evaluated by *in vivo* and *in vitro* experiments. It was found that the drugs wrapped by AOBs could be selectively internalized by tumor cells during the delivery process with up to 90 % internalization efficiency, which suggested strong anti-tumor activity. Such results demonstrate the great potential of AOBs as carriers of hydrophobic drugs in targeted drug delivery (Chiang et al., 2011, Chiang et al., 2018).

### Functional food emulsions

AOBs are frequently employed for encapsulating functional substances in the food industry. The oil phase of AOBs can dissolve hydrophobic compounds effectively, offering greater biocompatibility and biosecurity compared to conventional emulsions. The unique structure and properties of oleosin at the AOB interface are the primary reasons for its role as the emulsifier. Unlike common protein emulsifiers, Oleosins are small-sized, with significantly large hydrophobic domains. This allows them to firmly anchor at the oil–water interface, forming a tight interface membrane with phospholipids (Rayner, 2015). This characteristic contributes to the chemical and physical stability of the emulsions. Besides, Sun et al., (2022) discovered that the oil absorption capacity of oleosins can reach 5.2 g/g through ultrasonic-assisted salt treatment. Previous study suggested that enhanced oil adsorption is effectively contributed to the smooth and silky mouthfeel perception of food products (Lu et al., 2023). Therefore, AOBs can also serve as natural stable emulsions in food items such as mayonnaise and ice cream.

#### Delivery of bioactive compounds

Many bioactive ingredients, such as β-carotene and curcumin, exhibit easy oxidation, low solubility in water and low bioavailability, limiting their use in food formulations (Meng et al., 2024). Ding et al. (2021) addressed this challenge by dissolving β-carotene in soybean oil and constructing AOB by incorporating oleosins and lecithin. Simultaneously, using the same method, they replaced oleosin proteins with soy protein isolate for comparison. The results revealed that the emulsion constructed with oleosin and lecithin demonstrated superior encapsulation efficiency. Moreover, during the *in vitro* digestion process, it exhibited higher cellular absorption and bioavailability. Similarly, Sun et al. (2023) utilized AOB for the encapsulation of curcumin, they discovered that the addition of oleosin reduced the size and interfacial tension of emulsion droplets. The small droplet size facilitated the transfer of curcumin into micelles, thus enhancing its bioaccessibility.

When deliver the bioactive molecules, the metabolomics study of AOBs in the human body is necessary. Through metabolomics analysis, the relationship between functional foods and health is assessed at different molecular levels, providing more effective information for the development of functional foods (Li et al., 2021). Compared with natural oil bodies, AOBs can be better used in food formulations by optimizing the composition to improve the metabolic properties. Currently, most studies focus on the effect of lipid composition on human metabolism. In general, the hydrophobic core in the preparation of AOBs is made of common vegetable oils, the main component of which is TAG. However, excessive intake of TAG can lead to its accumulation in fat cells, resulting in obesity. In contrast, diacylglycerol (DAG) has a stronger ability to inhibit fat accumulation than TAG (Yu et al., 2017). Lu et al. (2020) found that DAG-enriched oil significantly reduced body weight and kidney weight as well as serum triglyceride levels in mice compared to TAG-enriched oil, which improved the lipid metabolism levels markedly. In addition, Liu et al. (2022) discovered that feeding mice with fish oil added high-fat diet reduced their body weight and alleviated the symptoms of dyslipidemia and hepatic steatosis. Overall, replacing TAG with DAG or adding fish oil to TAG are both effective methods to improve lipid metabolism in AOBs.

#### Prevents oxidative deterioration of oil

Replacing saturated fatty acids with unsaturated fatty acids in daily life may play an important role in reducing the likelihood of cardiovascular disease (Mozaffarian, 2011). However, foods containing high concentrations of unsaturated fatty acids are highly susceptible to deterioration and oxidation during both processing and storage. Oil bodies, as organelles for storing TAGs, have excellent physical stability and separate entities that do not aggregate when subjected to certain external pressures and can maintain their structural integrity (Huang, 1992, Tzen and Huang, 1992). Compared to emulsions stabilized with surfactants, oil bodies dispersed in the aqueous phase have a higher antioxidant capacity (Fisk et al., 2008). Inspired by the structure and function of natural oil bodies, more and more researches begin to encapsulate unsaturated fatty acids by forming AOBs, which are far more popular with consumers than adding additional antioxidants to prevent the oxidation of unsaturated oils, due to their green and safe nature. Wijesundera et al. (2013) extracted the OBP from canola to stabilize the AOBs, and then homogenized the mixture of tuna oil, OBP, and PL in a ratio of 10/1.5/0.1. By further studying the oxidative stability of the emulsion, the results confirmed that AOB encapsulated unsaturated fatty acids has great oxidative stability because of the unique amphiphilic structure of the OBP. In addition, a comparison of tween 40 emulsified tuna oil with AOBs encapsulated tuna oil under accelerated oxidation conditions revealed that the oxidative stability of AOB emulsion was significantly higher (Wijesundera & Shen, 2014). Besides, the addition of tocopherol to the emulsion is also an effective measure to enhance the antioxidant property and the chemical stability (Teixeira et al., 2017). Feng et al. (2020) prepared tocopherol contained nano-emulsion and added it to fish sausages. They found that the peroxide value of the fish sausages could be significantly reduced, as well as the emulsion was well dispersed with small particle size. These studies demonstrate the promising application of AOBs combined with tocopherols in preventing the oxidation of unsaturated oils in the food industry.

### Sensory evaluation

Tea beverages are widely loved by people all over the world because they can satisfy consumer’s demands for health, which are attributed to the presence of caffeine, catechins and flavonol glycosides (Shi et al., 2021). During the tasting of tea beverages, the main sensory feature is astringency, as polyphenols can combine with salivary proteins to form polymers to stimulate the oral epithelial mucosa, resulting in insufficient lubrication of saliva (Linne & Simons, 2017). Nowadays, there are many oversimplified approaches to evaluating astringency, however, these methods are not satisfying in the actual determination process. To precisely assess the astringency of tea beverages, more and more researchers are committed to promoting the approach in order to accelerate its use at the industrial level. Up to today, researchers have developed AOBs that are modified by recombinant caleosin fused with histatin 3 (a peptide from human salivary) to evaluate the relative astringency of tea beverages. Compared to conventional strategies, the use of AOBs offers a more convenient option, as the thickness of emulsion formed by the addition of tea can be used to determine the level of astringency of tea beverages (Shih et al., 2017). Meanwhile, the thickness of the emulsion is proportional to the astringency. Based on this, Liu and Tzen (2022) further investigated the astringency of the eight tea catechins utilizing AOBs, offering the possibility of their application in industry.

## Measures to improve AOBs

**Fig 6:**
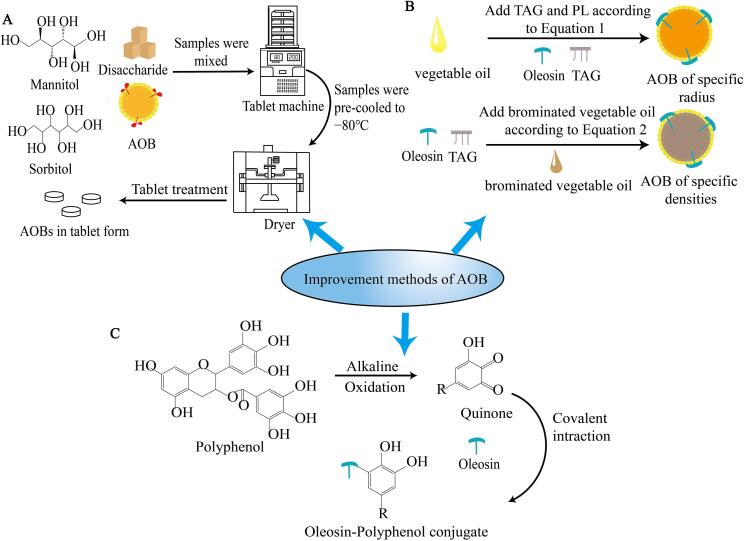
Improvement methods of AOBs. (A): Process for curing AOB. (B): Geometric framework for the reconstruction of AOB of specific sizes and densities. Equation1: Molar ratio of TAG: $\text{PL}=\frac{A_{\mathrm{PL}}N_{A}r_{\mathrm{LD}}\rho_{\mathrm{TAG}}}{3M_{\mathrm{TAG}}}$; Equation 2: $\%\text{BVO}=\frac{\rho_{\mathrm{desired}}-\rho_{\mathrm{TAG}}}{\rho_{\mathrm{BVO}}-\rho_{\mathrm{TAG}}}$. (C): Mechanism of oleosin modification by polyphenols.

Currently, the application of AOB is limited due to the short lifespan, uneven dispersion in the liquid and inadequate thermal stability. In order to improve the defects of the AOB, researchers have carried out relevant studies on the regulation of its particle size and density, as well as the preparation of new formulations. Furthermore, they explored the use of antioxidants to modify oil body membrane proteins to further improve its stability and efficacy. These efforts aim to promote AOB to be suitable for a variety of industrial applications (Fig. 6).

### Solidification of AOBs

Although the use of AOBs to encapsulate drugs can maintain their relative stability, it is still difficult to use them in practical production today because traditional forms of drug storage are predominantly solid tablets. Compared to the liquid form, the lower water content of solid tablets prevents the growth of micro-organisms and makes it more stable and convenient to package for transport. Therefore, a reasonable approach to modify AOBs into solid tablet form is key to overcome its inability to be produced industrially. Currently, Chang et al. (2013a) incorporated excipients such as sucrose, sorbitol, lactose, and mannitol into a blend of natural oil bodies and AOBs, which were subsequently subjected to drying and tablet compression. It was found that 90 % (w/w) mannitol in the excipient could effectively prevent oil body from aggregating and make it have a better curing effect. Besides, both AOBs and natural oil bodies remained stable after four months of storage without significant deliquescence. This convenient way of encapsulating and storing drugs by solidifying AOBs offers the potential for further commercial applications.

### Reconstitution of AOBs with specific size and density

The process of constructing AOBs is now well established, but it is not suitable for practical applications because the resulting AOBs are not uniformly dispersed in the emulsion and their size is difficult to control. To overcome these problems, Julien et al. (2021) developed a geometric framework to adjust the ratio of oil to PL to control the size of the AOBs and increase the central density of the oil body. The key to control the particle size of the oil is to adjust the ratio of TAGs and PLs. The formula for determining the size of the oil is as follows. In order to create AOBs of a specific radius, the molar ratio of TAG to PL can be calculated by substituting into Eq1.

(1) $$\mathrm{MolarratioofTAG}:PL=\frac{A_{\mathrm{PL}}N_{A}r_{\mathrm{LD}}\rho_{\mathrm{TAG}}}{3M_{\mathrm{TAG}}}$$

where A_PL_ is the surface area of the PL (the surface area of different types of PLs varies), N_A_ is Avagadro's number (6.022 × 1023 mol^−1^), $r_{\mathrm{LD}}$ is the radius of the AOBs, $\rho_{\mathrm{TAG}}$ is the density of the TAG, and $M_{\mathrm{TAG}}$ is the molecular weight of the TAG.

The production of AOBs with specific density requires the substitution of brominated vegetable oil (BOV) for normal vegetable oil, where the key point is to calculate the percentage of the addition of BVO (Eq.2). Based on the value of the target central density, the amount of brominated vegetable oil to be added can then be calculated.

(2) $$\%BVO=\frac{\rho_{\mathrm{desired}}-\rho_{\mathrm{TAG}}}{\rho_{\mathrm{BVO}}-\rho_{\mathrm{TAG}}}$$

$\rho_{\mathrm{desired}}$ represents the target central density, $\rho_{\mathrm{TAG}}$ represents the density of TAGs and $\rho_{\mathrm{BVO}}$ represents the density of brominated vegetable oil.

The creation of AOBs with a specific size and neutral buoyancy can significantly improve their efficiency in practical experiments. For example, microstructural observation of oil bodies requires which to be deposited on coverslips rather than floating in solution, and small particle sizes could facilitate spectroscopic measurements.

### Modification of proteins with antioxidants

AOB is difficult to store under natural conditions because the protein membrane is easy to be destroyed, resulting in flocculation of the oil body. Therefore, antioxidants such as polysaccharides and polyphenols can be added to modify the protein to improve its ability and resist the effects of environmental externalities (Liu et al., 2019). It was found that anthocyanins can modify proteins as well as change their structure and physicochemical properties (Chen et al., 2019, Chen et al., 2019). Similarly, Epigallocatechin-3-gallate (EGCG) in green tea, a polyphenol with high activity, not only has antioxidant and anticancer effects, but also can be added to proteins to obtain more porous structures, thus improving many of their properties, including antioxidant properties, emulsification and thermal stability (Huang et al., 2022, Yan et al., 2021). Sun et al. (2021) first fully hydrated the oil-body proteins and then added different concentrations of EGCG to covalently couple with that to form conjugates. It was found that the coupling not only resulted in more homogeneous emulsions with enhanced thermal stability but also better retention of curcumin activity, which provides a new idea for the use of oil bodies to deliver hydrophobic drugs.

#### Current challenges

Emulsions are not composed of a single component, but rather a complex contains multiple compounds. These complexes could improve interfacial properties by adsorbing amphiphilic emulsifiers to change the hydrophobicity of the surface (Wei et al.,2019). AOBs also have the problem of oil droplet flocculation due to the possible disruption of the membrane at the oil droplet interface during long-term storage (Qi et al., 2017). Therefore, for practical applications in the food industry, the complexes of the emulsions can be altered to explore the improvement of the environmental stability and sensory properties of AOBs in food formulations through the addition of hydrocolloids with higher biocompatibility and lower toxicity (Wang & Zhao, 2022). Currently, the physical stability of oil-in-water Pickering emulsions have been improved by adding xanthan gum, carrageenan, and hydroxypropyl methylcellulose (Xiao et al., 2021, Ren et al., 2021, Li et al., 2021, Yan et al., 2021, Zhong et al., 2022, Hu et al., 2022). The addition of these complexes can be used for the subsequent study of AOBs.

While AOB is composed of natural ingredients, its potential allergenic and toxic properties cannot be overlooked. Currently, oleosins from peanuts, sesame, and hazelnuts have been registered as allergens, and it may induce allergic reactions in individuals with thinner skin (Jappe & Schwager, 2017). Furthermore, the manufacturing processes of food can influence the allergenicity of oleosins. Mondoulet et al. (2005) found that the allergenicity of oleosin is heightened during the baking process, requiring manufacturers to label their products with allergens and improve their production processes to avoid harming consumer health.

Currently, AOBs face various challenges in practical applications, including crucial issues such as enhancing stability and addressing safety concerns in food formulations. To address these challenges, we need to increase research investment and consistently innovate to better advance the application of AOB technology.

#### Future perspectives

The design of transport carriers for loading, delivering and releasing functional ingredients has been a major research direction in the food and pharmaceutical industries (Light & Karboune, 2021). AOBs, as carriers of bioactive ingredients, not only can enhance the bioavailability and stability of the encapsulated substances, but also allow the design of the composition and size according to their characteristics. Therefore, AOBs can be tailored to meet the needs of applications in different fields, such as medicine, food science, agriculture, and so on. In fact, in food production, which often requires high-temperature processing procedures, the desirable and acceptable qualities of food products were highly dependent on the heat transfer properties (Ran et al., 2023). For oil-in-water emulsions, heat transfer properties are enhanced by selecting high viscosity oils and emulsions with relatively large particle sizes (Kumano et al., 2017, Nomura and Kumano, 2018). Therefore, the AOBs with precisely adjusted size also have great potential in the application for food processing and could serve as potential materials for food storage. When AOBs are practically applied in the food industry, we cannot ignore the preparation cost and the nutritional metabolism research *in vivo* and *in vitro*. However, at present, very few studies are conducted in the related field, and there is an urgent need to invest more efforts.

## Conclusion

Researches on AOBs has been carried out for decades, and still remain a hot topic due to the great exploitation value of which in food and pharmaceuticals. Currently, researches on AOBs mainly focused on enhancing their stability and expanding their applications. A large number of studies have shown that AOBs, as carriers for transporting functional substances, exhibit superior performance in terms of physicochemical stability, biocompatibility, and bioaccessibility. Similarly, in the field of biotechnology, AOBs can be used as carriers for purification, renaturation and immobilization of proteins, which greatly shortens the traditional process and improves the production efficiency. In summary, AOBs demonstrate versatile functionalities, holding great development potential in the fields of food, pharmaceuticals, and biotechnology.

## CRediT authorship contribution statement

**Ruhuan Yuan:** Conceptualization, Data curation, Formal analysis, Visualization, Writing – original draft, Writing – review & editing. **Jianying Liu:** Data curation, Formal analysis, Writing – original draft. **Ruchika Hansanie Ukwatta:** Data curation, Formal analysis. **Feng Xue:** Conceptualization, Writing – review & editing. **Xiaohui Xiong:** Funding acquisition, Project administration. **Chen Li:** Funding acquisition, Supervision, Writing – review & editing.

## Declaration of competing interest

The authors declare that they have no known competing financial interests or personal relationships that could have appeared to influence the work reported in this paper.

## Data Availability

No data was used for the research described in the article.
